# Physical Development and Postural Behaviors in Children and Adolescents: A Cross-Sectional Study

**DOI:** 10.3390/children13050698

**Published:** 2026-05-19

**Authors:** Francesca D’Elia, Rosario Ceruso, Giuseppe Giardullo, Angelica Delfina Picone, Vera Simoes, Tiziana D’Isanto, Giovanni Esposito

**Affiliations:** 1 Department of Human, Philosophical and Education Sciences, University of Salerno, 84084 Fisciano, Italy; fdelia@unisa.it (F.D.); a.picone8@studenti.unisa.it (A.D.P.); 2 Research Centre for Physical Education and Exercise, Pegaso Telematic University, 80143 Naples, Italy; rosario.ceruso@univr.it (R.C.); tiziana.disanto@istruzione.it (T.D.); giovanniesposito410@gmail.com (G.E.); 3 Department of Neuroscience, Biomedicine and Movement, University of Verona, 37129 Verona, Italy; 4 School of Sport Rio Maior (ESDRM), Santarém Polytechnic University—Santarém & Rio Maior, 2001-904 Santarém, Portugal; verasimoes@esdrm.ipsantarem.pt

**Keywords:** musculoskeletal pain, back pain, developmental age, neck pain, associations

## Abstract

**Highlights:**

**What are the main findings?**

- Flexed and asymmetrical sitting postures during academic and informal activities were significantly associated with back pain in children and adolescents.
- Gender differences emerged, with girls reporting a higher prevalence of back pain, while lifestyle factors (physical activity, screen time, sleep) showed no significant associations.

**What is the implication of the main finding?**

- The results identify daily postural habits as modifiable behavioral determinants of adolescent musculoskeletal pain, expanding current theoretical models that emphasize multifactorial interactions.
- Findings support the development of targeted school-based interventions, including posture education, ergonomic adjustments, and structured movement breaks, to reduce cumulative spinal loading during adolescence.

**Abstract:**

**Background/Objectives:** Musculoskeletal pain represents a frequent and increasingly recognized health issue during adolescence. During this developmental phase, daily habits such as prolonged sitting, sedentary behaviors and the adoption of non-neutral postures may contribute to spinal overload and pain symptoms. This study contributes to the existing literature by focusing on concrete postural behaviors in real-life daily situations, an aspect that is still underexplored despite its relevance for musculoskeletal health. The aim of this study was to investigate the associations between daily habits, postural behaviors and the presence of back and neck pain. **Methods**: A cross-sectional study was conducted on a sample of 70 adolescents aged 10–20 years. Data were collected using the Back Pain and Body Posture Evaluation Instrument for Children and Adolescents (BackPEI-CA), administered online. Descriptive statistics and chi-square tests with effect sizes (Cramer’s V) were used to examine associations between postural behaviors, lifestyle factors, and pain outcomes, providing an inferential assessment beyond simple descriptive analysis. **Results**: Significant associations were found between gender and back pain, sitting posture during daily activities (writing at the desk and talking with friends) and back pain, with flexed or asymmetrical postures more frequently reported among participants with pain, and between back and neck pain (*p* < 0.05). **Conclusions:** The findings highlight the relevance of gender and daily postural behaviors as factors associated with musculoskeletal pain in adolescents. By identifying posture-specific behaviors linked to pain, this study provides preliminary evidence that can inform targeted preventive strategies and guide future research on modifiable daily habits in youth.

## 1. Introduction

In recent decades, profound changes in the lifestyles of children and adolescents have raised growing concerns in the field of public health [1]. The increase in sedentary lifestyles, combined with the increasingly widespread use of digital devices, has led to a significant reduction in opportunities for engaging in daily physical activity [2], with potential repercussions for the harmonious development of the musculoskeletal system [3]. This trend aligns with research showing that motor development and biomechanical efficiency are closely linked to learning processes and movement quality throughout growth [4,5]. In this context, posture during childhood and adolescence is particularly important, not only as an expression of body alignment but also as an indicator of overall health and psychophysical development [6]. Childhood, which extends from birth to late adolescence [7,8], is a phase characterized by intense processes of growth, maturation and development and it needs targeted educational activities to enhance psychophysical development [9]. In line with this perspective, the present study focuses on a developmental range spanning from late childhood to adolescence and early adulthood (10–20 years), a period in which school-related sedentary behaviors, independent use of digital devices, and postural behaviors become increasingly consolidated. Although this range spans multiple developmental stages, it reflects the age distribution of the available sample and is interpreted with appropriate caution. During this period, the body undergoes profound structural and functional transformations in the muscular, skeletal and nervous systems, making posture a dynamic and constantly evolving phenomenon [10]. The physical activity and postural behaviors acquired during this phase tend to consolidate over time, exerting a lasting influence on future well-being [11,12,13]. This perspective aligns with findings showing that teaching methods can significantly influence children’s motor efficiency, perceptions and awareness [14]. For this reason, any postural deviations that arise during childhood and adolescence are of primary clinical and preventive importance. Postural deviations during childhood may initially manifest as poor posture or paramorphism, i.e., functional and reversible alterations in posture that do not yet involve permanent structural changes to the spine. However, in the absence of early and targeted intervention, these conditions can evolve into dysmorphisms, characterized by irreversible structural alterations, such as scoliosis, hypokyphosis and hyperlordosis [15,16]. These conditions are increasingly common among children and adolescents, as shown by evidence indicating a rise in posture-related and cervical musculoskeletal disorders associated with smartphone use [17], often accompanied by musculoskeletal pain, functional limitations and, in severe cases, respiratory impairment and reduced quality of life [18].

Epidemiological studies have highlighted a high prevalence of postural deviations among children and adolescents, with a particularly elevated incidence during school-age years [19,20]. These deviations are often subtle and initially asymptomatic, making timely diagnosis difficult. This “clinical invisibility” allows the alterations to progress silently over time [13]. Furthermore, poor posture is associated not only with physical consequences, but also with psychological effects, influencing self-esteem, body image and socialization processes, especially during adolescence, a sensitive phase for identity development [21]. From a theoretical perspective, posture cannot be reduced to a fixed “correct position” or ideal alignment. The notion of a single postural standard has been increasingly questioned, as current evidence shows that so-called “ideal alignment” is based on outdated anatomical assumptions, does not account for natural interindividual variability, and may lead to misleading classifications of postural deviations, particularly when compared with natural postural patterns observed across ages and populations [22]. This perspective reinforces the need to interpret posture as a dynamic, individualized phenomenon rather than a configuration to be matched against a universal standard. Scientific literature describes posture through complementary interpretative models, including neurophysiological, biomechanical and psychosomatic ones, which together offer an integrated view of postural control [23]. In this framework, the emphasis shifts from achieving a predefined “ideal” posture to understanding how individuals naturally adapt their alignment to maintain balance, minimize energy expenditure and respond to internal and external stimuli. The neurophysiological model emphasizes the role of the nervous system and proprioception in regulating posture [24]; the biomechanical model focuses on mechanical forces, load distribution and movement efficiency [25]; finally, the psychosomatic model highlights the relationship between posture, emotional states and psychological well-being [10]. The integration of these approaches is fundamental to a complete understanding of postural problems in childhood.

This broader conceptualization aligns with recent discussions questioning whether deviations from traditional postural standards necessarily indicate dysfunction. Although correlations between postural irregularities and pain have been reported, evidence remains inconsistent, and natural compensatory patterns, especially those emerging with age, may be misinterpreted as pathological when compared to unrealistic postural ideals [22].

At the same time, modern lifestyles have introduced additional risk factors affecting postural development. Sedentary behaviors, characterized by minimal energy expenditure, are now extremely common among young people. Long periods spent sitting, often in non-ergonomic environments such as classrooms or at home, encourage non-neutral and repetitive postural behaviors [26,27]. Prolonged use of electronic devices, such as smartphones, tablets and computers, further contributes to non-physiological postures, increasing spine and cervical load, representing a risk factor for musculoskeletal discomfort and obesity [28,29,30]. These behavioral patterns interact with natural postural variability, potentially reinforcing compensatory strategies that, although functional in the short term, may become maladaptive when combined with prolonged static positions.

In school settings, this issue becomes even more relevant, as students spend many hours a day seated at their desks. The ergonomics of school furniture, the organization of lessons and the absence of active breaks are recognized as key factors influencing postural behaviors. Evidence shows that inadequate sitting postures during school activities, such as sitting or writing at a desk, are significantly associated with structural changes in the spinal column, including alterations in thoracic kyphosis, lumbar lordosis and scoliosis, underlining the impact of school-related behaviors on postural development [31]. Previous studies have shown that poorly designed school environments can increase the incidence of musculoskeletal disorders and negatively affect students’ overall well-being [32]. Conversely, research on heuristic learning highlights how active, exploratory and student-centered approaches can promote healthier motor patterns and postural behaviors [33,34].

Considering this evidence, it is clear that the prevention of postural problems must be based on an early, comprehensive and multidisciplinary approach involving the family, school and health and movement professionals. Postural education and the promotion of an active lifestyle are fundamental tools for promoting harmonious development and preventing the onset of structural alterations of the spine [35]. Despite extensive theoretical and epidemiological literature on postural deviations in youth, empirical evidence directly examining how specific everyday postures contribute to these alterations remains limited [36]. Most existing studies focus on structural outcomes or general lifestyle indicators, while fewer investigations have analyzed the concrete postural behaviors assumed during study, leisure time or prolonged sedentary tasks, and how these behaviors interact with the natural variability of spinal alignment in real-life contexts [37]. Recent methodological studies have also highlighted the limitations of traditional static assessments, showing that many of the visual tools commonly used in clinical and educational settings do not fully reflect contemporary scientific understanding of posture [38]. The development of more holistic instruments demonstrates progress but also underscores the need for assessment approaches capable of capturing natural postural variability and the dynamic behaviors in adolescents’ daily lives. These findings further confirm that current evidence remains insufficient to clarify how everyday postural habits contribute to the development of spinal deviations.

Even though there is a substantial body of literature on postural deviations and musculoskeletal pain in youth, important gaps remain. Most existing studies have focused on structural spinal alterations or general lifestyle indicators, whereas the specific postural behaviors adopted during everyday activities, such as studying, interacting with peers, or using digital devices, have been examined far less frequently. Moreover, many investigations rely on static or idealized postural assessments that do not fully capture the dynamic and context-dependent nature of posture in real life. As a result, the contribution of concrete daily postural habits to musculoskeletal symptoms remains insufficiently clarified. Addressing these gaps is essential, as adolescence is a developmental period in which sedentary routines, school-related sitting, and digital media use become increasingly consolidated and may interact with natural postural variability. Understanding how these behaviors relate to pain can provide valuable insights for prevention and health promotion.

Therefore, the present study aims to investigate the associations between daily lifestyle factors, postural behaviors, and the presence of back and neck pain in a sample of children and adolescents. To guide this investigation, the study addresses the following research questions:

RQ1. Are specific postural behaviors adopted during daily activities (e.g., writing at the desk, talking with friends, using digital devices) associated with the presence of back or neck pain?
RQ2. Are lifestyle factors (e.g., screen time, sleep habits, physical activity) associated with musculoskeletal pain in this population?
RQ3. Are there gender-related differences in the prevalence of pain and in the postural behaviors reported?

## 2. Materials and Methods

### 2.1. Study Design

A cross-sectional study was conducted to investigate back pain, postural behaviors, and lifestyle factors associated with spinal health in children and adolescents. The study is part of the broader research project “Psycho-physical perception and body awareness in school and sports contexts through observational and non-invasive tools”, approved by the Ethics Committee of Pegaso University (Prot./E 004726, 15 July 2025). All procedures adhered to the principles of the Declaration of Helsinki.

### 2.2. Participants

The study involved a sample of 70 participants, of whom 33 were female (47%) and 37 male (53%). Participants ranged in age from 10 to 20 years, with a mean age of 15 ± 2.17 years. The age range of 10–20 years was selected to include late childhood and adolescence, developmental phases characterized by school attendance, prolonged sitting, and independent digital device use. This range also reflects the extended transition from adolescence to early adulthood described in recent developmental frameworks [7,8]. Although this span includes multiple developmental stages, analyses were conducted with appropriate caution.

Average body weight was 62 ± 14.03 kg, and the average height was 167 ± 9.86 cm.

All participants, or their parents or legal guardians when required, provided informed consent prior to inclusion in the study.

Participants first completed an introductory section collecting demographic information (age, sex, weight, height), followed by the administration of the BackPEI-CA questionnaire. To ensure sample homogeneity, inclusion criteria required participants to be aged between 10 and 20 years, regularly attending school, and without diagnosed neurological, orthopedic or rheumatologic conditions that could affect posture or pain perception. Exclusion criteria included recent musculoskeletal injuries (within the previous three months), ongoing physiotherapy treatment, or incomplete questionnaire responses. Participants were recruited through convenience sampling in local schools and sports associations, and participation was voluntary. No missing data were detected in the final dataset, as the online platform required completion of all items before submission.

The relatively small sample size should be considered when interpreting the generalizability of the findings.

### 2.3. Assessment Tool: The BackPEI-CA

The BackPEI-CA is a validated questionnaire designed to assess back pain, neck pain, postural behaviors, and lifestyle factors in children and adolescents [39,40]. It includes male and female versions; both share identical content and structure, differing only in layout and illustrations.

The questionnaire includes 30 items, of which 28 are multiple-choice and 2 (items 25 and 30) use a Visual Analogue Scale (VAS) to measure pain intensity [40]. The instrument covers four main domains:

- lifestyle and daily habits (e.g., physical activity, screen time, sleep habits);
- postural habits, assessed through illustrated figures depicting common postures during studying, computer use, mobile device use, lifting objects, and carrying backpacks;
- back pain, including presence, frequency, duration, functional limitations, and VAS intensity;
- neck pain, assessed with the same structure as back pain;

Examples from the questionnaire include:

- “How many hours per day do you usually spend sitting watching television?”
- “How do you typically sit at your desk when writing while in school?”
- “On the scale from 0 to 10, please identify the intensity of your back pain for the last 3 months.”

The BackPEI-CA was administered in Italian. Because no validated Italian version existed, the research team performed a simplified cross-cultural adaptation procedure. Two bilingual researchers independently translated the questionnaire (forward translation), and discrepancies were resolved through consensus. A third expert in exercise sciences reviewed the final version to ensure semantic and conceptual equivalence. A pilot test with five adolescents confirmed clarity and comprehensibility of items and illustrations. Although adapted linguistically, a full psychometric validation of the Italian version was not conducted prior to data collection, and this should be considered when interpreting the results. The predominance of image-based items, however, substantially reduced the potential impact of linguistic bias.

### 2.4. Procedure of Administration

The BackPEI-CA was administered via the Google Forms platform, allowing accessible and remote data collection. Participants received a link via email or messaging platforms, along with instructions for completion and the option to request clarification. Estimated completion time was approximately 15–20 min, with the possibility to pause and resume. The online format enabled intuitive presentation of items and illustrations.

### 2.5. Statistical Analysis

Statistical analyses were conducted using both descriptive and inferential approaches. Absolute frequencies (F) and percentages (%) were calculated for all items. Associations between categorical variables, specifically perceived pain (back or neck pain) and postural or lifestyle behaviors, were examined using a chi-square test (χ^2^). When significant associations were found, Cramer’s V was calculated to quantify effect size.

The level of statistical significance was set at *p* < 0.05. No correction for multiple testing was applied, as the analyses were exploratory and aimed at identifying potential associations; therefore, the results should be interpreted with caution due to the increased risk of type I error.

Given the limited sample size and the exploratory design, no multivariable models or confounder adjustments were performed.

All analyses were conducted using SPSS version 28.0 (IBM Corp., Armonk, NY, USA).

## 3. Results

Table 1 summarizes participants’ characteristics and the presence of back and neck pain. Regarding screen-based behaviors, 51% of participants reported using smartphones or tablets for more than 4 h per day, while 27% reported 2–3 h/day. Computer use and TV watching were less frequent. Sleep duration averaged 7.2 ± 1.1 h per night. The prevalence of back pain in the previous three months was 63% (*n* = 44), with a mean VAS intensity of 4.08 ± 1.83. Neck pain was reported by 56% (*n* = 39), with a mean VAS intensity of 3.92 ± 2.20.

Table 2 presents the distribution of postural behaviors adopted during daily life. Flexed trunk and asymmetrical sitting postures were the most frequently reported patterns across different contexts, including writing at the desk, computer use, and smartphone/tablet use. Upright sitting postures were less commonly reported, particularly during smartphone and tablet use, where only a small proportion of participants reported maintaining an upright posture. When using a smartphone or tablet while standing, postural behaviors characterized by moderate to marked neck flexion predominate, whereas upright standing posture with the device held at eye level was rarely reported. Similarly, when picking up objects from the floor, trunk-flexion strategies were reported more frequently than squatting postures.

A statistically significant association with a moderate effect size was observed between gender and the presence of back pain in the previous three months (χ^2^ test, *p* = 0.021; Cramer’s V = 0.31). Female participants reported a higher prevalence of back pain compared with male participants. A detailed description is provided in Table 3.

A statistically significant association with a moderate-to-large effect size was observed between sitting posture at the desk while writing and back pain reported in the previous three months (χ^2^ (3) = 12.62, *p* = 0.004; Cramer’s V = 0.42). A higher prevalence of back pain was observed among participants reporting a flexed trunk posture, whereas lower prevalence was reported among those adopting upright or asymmetrical sitting postures. A detailed description is provided in Table 4.

A statistically significant association with a moderate-to-large effect size was observed between sitting posture while talking with friends and the presence of back pain in the previous three months (χ^2^ test, *p* = 0.003; Cramer’s V = 0.45). A higher prevalence of back pain was observed among participants reporting flexed or asymmetrical sitting postures. A detailed description is provided in Table 5.

Overall, the effect sizes indicated associations of moderate (V = 0.31) to moderate-to-large magnitude (V = 0.42–0.45). These values suggest that the identified relationships, although not explanatory in a causal sense, reflect meaningful patterns in the data, particularly regarding posture-related behaviors during daily activities. However, the magnitude of these associations should be interpreted with caution given the exploratory design and the limited sample size.

## 4. Discussion

The findings of this study contribute to the growing body of evidence on musculoskeletal pain in adolescents, highlighting the relevance of gender and daily postural behaviors in relation to back and neck symptoms. Although several lifestyle factors did not show significant associations with pain, the results underscore the multifactorial nature of musculoskeletal discomfort during adolescence and the importance of considering both biomechanical and behavioral dimensions. From a theoretical perspective, these associations may be interpreted through biomechanical models showing that sustained flexed or asymmetrical postures increase intervertebral disc pressure, modify muscle activation patterns, and reduce postural variability [41]. These mechanisms may help contextualize the observed associations, although the cross-sectional design prevents any inference regarding causality. Behavioral mechanisms may also play a role, as habitual postural strategies adopted during academic and social activities can become consolidated patterns that influence comfort and pain perception over time. Students’ perceptions of the impact of sport participation on their well-being may further influence the adoption of healthier motor and postural behaviors, as shown in recent school-based investigations [42].

### 4.1. Gender and Back Pain: Confirmation of Consolidated Evidence

A significant association emerged between gender and the presence of back pain, with female participants reporting a higher prevalence of symptoms. This finding is consistent with previous research showing that musculoskeletal pain is more frequently reported by adolescent girls than boys [43]. Although several biological, psychosocial, and behavioral explanations have been proposed, the moderate effect size observed in this study (Cramer’s V = 0.31) indicates that gender accounts for only part of the variability in pain outcomes [6,44]. From a biomechanical perspective, gender-related differences in spinal morphology may play a role. Evidence indicates that females tend to present greater lumbar lordosis and thoracic kyphosis compared with males, variations that may influence spinal loading during daily activities and predispose girls to discomfort during prolonged sitting or flexed postures [15,16]. These morphological characteristics have been observed even in school-aged populations and may interact with lifestyle factors to increase susceptibility to pain.

Behavioral aspects also contribute to this disparity. Studies examining sedentary behavior and musculoskeletal symptoms in adolescents have shown that girls often accumulate more sedentary time and engage less frequently in vigorous physical activity, factors associated with increased musculoskeletal discomfort [12]. In particular, da Costa et al. reported that sedentary behavior was significantly associated with both neck and low back pain in girls, whereas in boys the association was limited to neck pain.

Although the association between gender and back pain was statistically significant, the effect size was moderate (Cramer’s V = 0.31), indicating that gender contributes meaningfully but only partially to the variability in pain outcomes. This aligns with the multifactorial nature of adolescent musculoskeletal pain, suggesting that gender interacts with other behavioral and biomechanical factors rather than acting as a dominant predictor.

### 4.2. Postural Behaviors During Daily Activities and Their Association with Back Pain

The study identified significant associations between specific postural habits adopted during daily activities and the presence of back pain. In particular, flexed or asymmetrical sitting postures while writing at the desk or interacting socially with friends were associated with a higher prevalence of pain. These findings suggest that certain daily postural behaviors may be relevant correlates of musculoskeletal discomfort, although they cannot be interpreted as causal contributors. The results should also be interpreted in light of contextual factors not assessed in this study, such as school ergonomics, furniture design, or movement breaks, which may influence postural behaviors. Although the present study did not directly assess school furniture, classroom ergonomics, or the implementation of active breaks, the previous literature suggests that these contextual factors may influence postural behaviors in school-aged populations. Our findings, which highlight the association between daily sitting postures and musculoskeletal pain, reinforce the need for future studies to examine how school environments and movement-based strategies may contribute to healthier postural habits [45]. These considerations should be interpreted as broader implications rather than conclusions derived from the variables measured in this study. Indeed, the moderate-to-large effect sizes (Cramer’s V = 0.42–0.45) indicate meaningful associations within the sample, but these values still explain only part of the variability in pain outcomes.

This interpretation is consistent with evidence showing that prolonged sitting in non-neutral postures increases mechanical stress on spinal structures and may contribute to musculoskeletal discomfort, especially when combined with insufficient postural variability and muscle fatigue. Several studies have emphasized how sedentary behaviors and repetitive flexed postures, common during school activities, computer use and smartphone use, can overload the cervical and lumbar spine [11,12,28]. These biomechanical effects are amplified when flexed postures are maintained for extended periods, as reduced activation of deep stabilizing muscles and increased reliance on passive structures (ligaments, fascia) may decrease postural endurance and heighten susceptibility to discomfort. This provides a plausible mechanistic explanation for the moderate-to-large associations observed in this study between flexed sitting postures and back pain. In particular, smartphone-related neck flexion has been identified as a relevant risk factor for cervical and upper-back symptoms in young populations [17].

The predominance of flexed trunk postures observed in this study across multiple contexts aligns with research documenting the widespread adoption of non-ergonomic postures among adolescents, especially during digital device use [31,36]. These behaviors may contribute to cumulative spinal loading, particularly when combined with prolonged static positions typical of school environments. Moreover, evidence from school-based studies indicates that inadequate ergonomics and poorly designed learning environments can exacerbate postural deviations and increase the incidence of musculoskeletal disorders [31,32].

The associations between posture-specific behaviors and back pain showed moderate-to-large effect sizes (Cramer’s V = 0.42–0.45), suggesting that these daily postural habits may play a non-negligible role in adolescents’ musculoskeletal discomfort. However, these values still account for only part of the variability in pain outcomes, reinforcing the need for analytical approaches capable of examining interacting predictors.

A key contribution of this study lies in its focus on posture-specific behaviors in real-life contexts, such as writing at the desk or interacting socially. While previous research has often examined general lifestyle indicators or structural spinal deviations, fewer studies have explored how concrete daily postures relate to pain. The present findings therefore add to existing knowledge by highlighting the relevance of everyday motor habits that may otherwise remain overlooked in traditional assessments.

### 4.3. Absence of Association Between Lifestyle Factors and Back Pain

The lack of significant associations between back pain and several lifestyle factors, including regular physical activity, screen time and sleep duration, is consistent with the idea that musculoskeletal pain in adolescence arises from complex interactions rather than isolated behaviors. However, the use of self-reported measures and the limited sample size may have reduced the sensitivity to detect such associations.

Recent evidence suggests that the type, intensity and motor quality of physical activity may be more relevant to musculoskeletal health than the mere amount of weekly exercise [9,13]. For example, activities that promote motor control, postural stability and movement variability may offer greater protective effects than general participation in sports.

Similarly, the absence of a significant association with screen time aligns with studies indicating that the postural strategies adopted during device use may be more influential than the duration of exposure itself. Research has shown that adolescents frequently adopt flexed or asymmetrical postures during smartphone and tablet use, which may contribute to spinal overload independently of total screen time [17,28]. This suggests that the biomechanical context of sedentary behavior, rather than its duration, may represent the primary factor influencing pain.

### 4.4. Relationship Between Back Pain and Neck Pain

The significant association between back pain and neck pain reflects patterns of co-occurrence frequently reported in adolescent populations. However, these findings indicate correlation rather than directionality, and longitudinal studies are needed to clarify temporal relationships. Moreover, postural deviations commonly observed in children and adolescents can affect global spinal alignment, reinforcing the interconnected nature of cervical and lumbar function [16,31]. These findings support the need for holistic preventive strategies that address the spine as an integrated system. It is important to emphasize that, due to the cross-sectional design, the associations identified cannot be interpreted causally. The findings indicate patterns of co-occurrence rather than directional effects, and longitudinal or experimental studies are needed to clarify temporal dynamics and underlying mechanisms.

Taken together, the findings highlight that musculoskeletal pain in adolescence arises from the interplay of biological, postural and behavioral factors. Gender differences, daily postural habits and the broader lifestyle context each contribute to shaping adolescents’ vulnerability to back and neck pain. These results should be interpreted as preliminary evidence of relevant associations rather than as indications for specific preventive actions.

The exclusive use of chi-square tests limited the analytical depth of the study and did not allow for the examination of structural relationships or the relative influence of different predictors. Nevertheless, the moderate-to-large effect sizes observed for posture-specific behaviors suggest that these associations warrant further investigation through multivariable models in larger samples. By integrating biomechanical, behavioral, and contextual perspectives, the present study contributes to a more nuanced understanding of adolescent musculoskeletal health and highlights modifiable daily habits that may serve as promising targets for future preventive strategies.

Future research should incorporate more detailed and objective assessments of physical activity, sedentary behavior, sleep and posture to better understand how these factors interact and to inform more targeted and effective interventions.

#### Limitations

This study presents several limitations that should be considered when interpreting the findings. The sample size was relatively small and based on convenience sampling, which limits the external validity of the results. Moreover, recruiting participants through local schools and sports associations likely introduced selection bias, resulting in a higher proportion of physically active adolescents than in the general population; this overrepresentation may further reduce generalizability, particularly regarding lifestyle habits and musculoskeletal symptoms. All data were self-reported, introducing potential recall bias, although the use of illustrated items likely reduced misinterpretation. The cross-sectional design prevents any inferences about causal relationships between posture, lifestyle factors and pain. Although BackPEI-CA was translated following a structured linguistic and cultural adaptation process, the Italian version was not formally validated prior to data collection, which may affect measurement reliability. Additionally, lifestyle variables such as physical activity, screen time and sleep were assessed through broad categories, which may not capture qualitative aspects relevant to musculoskeletal health. The analytical approach relied on chi-square tests without adjustment for potential confounders, due to the exploratory design and limited sample size. Furthermore, multiple chi-square tests were conducted without correction for multiple comparisons; while appropriate for an exploratory study, this increases the likelihood of type I error. For these reasons, the findings should be interpreted with caution as preliminary and hypothesis-generating rather than definitive.

## 5. Conclusions

This study highlights the multifactorial nature of musculoskeletal pain in adolescence, showing that gender differences, daily postural behaviors and the broader behavioral context are relevant correlates of back and neck pain. Flexed and asymmetrical postures adopted during both academic and informal activities were associated with a higher prevalence of symptoms, while female participants reported greater susceptibility to back pain. These associations should be interpreted cautiously and do not imply causal relationships. The findings suggest that posture-related behaviors may represent meaningful areas for further investigation, although the present data do not allow conclusions regarding preventive strategies. Future research should employ longitudinal designs and integrate objective assessments of posture, physical activity and sedentary behavior to better understand the complex interactions underlying adolescent musculoskeletal health.

Regarding the three research questions, the study showed that posture-specific behaviors were meaningfully associated with pain (RQ1), lifestyle indicators did not demonstrate significant relationships (RQ2), and gender differences emerged as a relevant factor (RQ3). This alignment reinforces the internal coherence of the study and clarifies its contribution to the existing literature. A distinctive contribution of this research lies in its focus on concrete, real-life postural behaviors, such as writing at the desk or interacting socially, rather than on general lifestyle indicators or structural deviations. By examining how adolescents position themselves in everyday contexts, the study offers insights into daily motor habits that may warrant further investigation. The findings also suggest that theoretical frameworks on adolescent musculoskeletal pain may benefit from a greater emphasis on habitual postural strategies and their interaction with developmental factors such as spinal maturation, motor control and sedentary routines. From a public health perspective, the results highlight areas that future school-based research may explore, although the present study does not provide evidence to support specific policy recommendations.

Overall, this study underscores the relevance of posture-specific behaviors as context-dependent correlates of adolescent musculoskeletal health, providing a preliminary foundation for future theoretical developments and hypothesis-driven preventive research.

## Figures and Tables

**Table 1:** Descriptive analysis of sample characteristics and presence of pain.

Question	Answer	F (%)
1. Practice sport/exercise	Yes	50 (71)
	No	20 (29)
2. Frequency of sport/exercise	1–2 days/week	22 (44)
	3–4 days/week	21 (42)
	≥5 days/week	5 (10)
	It varies	2 (4)
3. Competitive sport	Yes	18 (36)
	No	32 (64)
4. TV watching time	0–1 h/day	38 (54)
	2–3 h/day	17 (24)
	4–5 h/day	1 (2)
	It depends on the day	14 (20)
5. Desktop/laptop use	0–1 h/day	40 (57)
	2–3 h/day	20 (29)
	≥4 h/day	10 (14)
6. Cellphone/tablet use	0–1 h/day	1 (2)
	2–3 h/day	19 (27)
	4–5 h/day	19 (27)
	≥6 h/day	17 (24)
	It depends on the day	14 (20)
7. Study/read in bed	Yes	27 (39)
	No	43 (61)
8. Favorite sleeping position	Face down	12 (17)
	Face up	9 (13)
	On the side	30 (43)
	It varies	19 (27)
9. Sleep duration	≤6 h	18 (26)
	7 h	27 (39)
	8–9 h	17 (24)
	≥10 h	5 (7)
	It depends on the day	3 (4)
21. Back pain in last 3 months	Yes	44 (63)
	No	24 (33)
	I do not know	3 (4)
26. Neck pain in last 3 months	Yes	39 (56)
	No	31 (44)
30. Pain intensity. Back pain (M = 4.08, SD = 1.83); Neck pain (M = 3.92, SD = 2.20)		

**Table 2:** Postural behavior adopted during daily life.

Question	Answer	F (%)
10. Sitting at desk while writing	A	31 (44)
	B	10 (14)
	C	18 (26)
	D	0 (0)
	E	0 (0)
	I could not identify one among these	11 (16)
11. Sitting while talking with friends	A	10 (14)
	B	28 (40)
	C	24 (34)
	D	0 (0)
	E	0 (0)
	I could not identify one among these	8 (12)
12. Sitting using computer	A	29 (41)
	B	13 (19)
	C	25 (36)
	D	0 (0)
	E	0 (0)
	I could not identify one among these	3 (4)
13. Sitting using phone/tablet	A	29 (42)
	B	20 (28)
	C	13 (19)
	I could not identify one among these	8 (11)
14. Using phone/tablet while standing	A	39 (56)
	B	26 (37)
	C	3 (4)
	I could not identify one among these	2 (3)
15. Picking up objects from the floor	A	14 (20)
	B	18 (26)
	C	13 (18)
	D	16 (23)
	I could not identify one among these	9 (13)

**Table 3:** Association between gender and back pain in the previous three months.

Gender	Back Pain: Yes	Back Pain: No	Not Sure	Total	χ^2^ Test (*p*)	V
Female	26	6	1	33	0.021	0.31
Male	18	17	2	37		

**Table 4:** Association between sitting posture at the desk while writing and the presence of back pain in the previous three months.

10. Sitting Posture at the Desk While Writing	Back Pain: No	Back Pain: Yes	Total	χ^2^ Test (*p*)	V
I could not identify one among these	5	6	11	0.004	0.42
A (Flexed trunk sitting posture)	7	24	31		
B (Upright sitting posture)	6	4	10		
C (Asymmetrical sitting posture)	13	5	18		

**Table 5:** Association between sitting posture while talking with friends and back pain in the previous three months.

11. Sitting Posture While Talking with Friends	Back Pain: No	Back Pain: Yes	Total	χ^2^ Test (*p*)	V
I could not identify one among these	6	2	8	0.003	0.45
A (Flexed trunk sitting posture)	0	10	10		
B (Upright sitting posture)	15	13	28		
C (Asymmetrical sitting posture)	10	14	24		

## Data Availability

The original contributions presented in this study are included in the article. Further inquiries can be directed to the corresponding author.
